# 

**Published:** 1910-12

**Authors:** 


					CONTENTS. Pagt
The Medical Aspect of BosweN's "Life of Johnson, with some
Account of the Medical Men mentioned in that Book. By
Bertram
M. H. Rogers, M.JJ., iS.Uti., tf.A.uxon.
289
Remarks on the Surgical Aspect of Glycosuria, and on Diabetic
Gangrene, with the Record of Three Cases in which Amputation
was performed for Gangrene in JDiabetics under Spinal Anses-
thesia.
By Charles A. Morton, I'.K.C.b.
3ii
Hyoscine and the Mydriatic Alkaloids.
By J. M. Fortescue-
Brickdale, M.A., M.D.Oxon.
317
On the Excision of Strictures of the Urethra.
By Ernest W. Hey
Groves, M.b.Lond., r.K.U.b.
325
An Old-Time Syphilidiater.
By Hubert J. Norman, M.tJ., u.r.hl....
334
Progress of the Medical Sciences :
Medicine.
George Parker, M.D., M.A. Cantab.
343
Surgery.
A. Rendle Short, M.D., B.S., B.Sc. L,ond.f r.K.C.b.
347
Obstetrics.
Walter C. Swayne, M.D. Lond....
352
neviews ot dooks
357
Editorial Notes
3^3
Notes on Preparations tor the sick
37?
The Library of the Bristol Medico-Chirurgical Society ... ... 374
Meetings of Societies
370
a* Obituary Notice
379
Local Medical Notes
3??

				

